# Relationship Between Companies' Responses to Near-Miss Reports and Turnover Intentions of Workers: A Nationwide Cross-Sectional Study

**DOI:** 10.1016/j.shaw.2024.01.004

**Published:** 2024-02-01

**Authors:** Ayaka Yamamoto, Tomohisa Nagata, Kiminori Odagami, Nuri Purwito Adi, Masako Nagata, Koji Mori

**Affiliations:** 1 Department of Occupational Health Practice and Management, Institute of Industrial Ecological Sciences, University of Occupational and Environmental Health, Japan; 2 Department of Community Medicine, Faculty of Medicine, Universitas Indonesia, Jakarta, Indonesia; 3 Department of Occupational Medicine, School of Medicine, University of Occupational and Environmental Health, Japan

**Keywords:** Near-miss, Response, Safety climate, Turnover intention, Workplace

## Abstract

**Background:**

Effective near-miss management is important in preventing workplace accidents. A company's inadequate response to near-miss reports can lead workers to feel insecure and dissatisfied with the company. We investigated the relationship between companies' responses to near-miss reports and turnover intentions of workers.

**Methods:**

We conducted a cross-sectional study using online self-administered questionnaire survey to workers aged ≥20 years in Japan in March 2022. The analysis included 5,071 participants who had near-miss experiences and reported them to their companies. The independent variable was companies' responses to near-miss reports, classified into three categories: adequate response group, inadequate response group, and no response group. The dependent variable was turnover intentions. We calculated the odds ratio and 95% confidential interval (CI) using multilevel logistic regression analyses nested for industries and adjusted for covariates.

**Results:**

Of the 5,071 participants, 3,058 (60.3%) were adequate response group, 1,484 (29.3%) were inadequate response group, and 529 (10.4%) were no response group. In multivariable adjusted model, compared with adequate response group, the odds ratio of inadequate response group and no response group were 1.80 (95% CI: 1.56–2.08) and 2.63 (95% CI: 2.15–3.22), respectively.

**Conclusion:**

Our results suggested that there was a relationship between companies' responses to the near-miss reports and turnover intentions of workers. It is important not only to collect near-misses but also to respond appropriately to the reports and provide feedback to workers.

## 1 Introduction

Effective near-miss management is important in preventing workplace accidents. The National Safety Council defines a near-miss as an unplanned event that does not result in injury or death but could have [1]. Heinrich's theory, established in 1929, states that behind each major accident, there are 29 minor accidents, and behind each minor accident, there are 300 near-misses [2,3]. Recent studies indicate that near-misses often precede incidents and managing near-misses effectively can reduce injuries [[3], [4], [5], [6]]. Therefore, when a near-miss event occurs, it is important that workers report the experiences to their companies and the companies take actions such as investigating the cause and improving equipment to prevent future accidents. Several studies have shown that safety activities using reporting near-misses, such as analyzing the cause of them and taking measures against them, can reduce the incidence of subsequent serious occupational accidents [4,7]. However, few studies have shown the adequacy of the company's responses to near-miss reports and how workers felt about the responses.

Ineffective safety management can intensify workers' turnover intentions. Turnover intentions often precede actual turnover behaviors, which are a significant concern for employers because of the economic impacts, such as productivity loss, elevated recruitment costs, increased training expenses, and so on [[8], [9], [10], [11]]. They are frequently used as indicators in health and safety studies [12,13]. One factor related to turnover intentions is safety climate, which indicates the extent to which workers perceive that safety is a priority at their company and a strong safety climate can reduce the likelihood of workers' turnover [[14], [15], [16], [17]]. Furthermore, active near-miss management has been shown to strengthen safety culture, which is conceptually similar to safety climate [18,19]. In short, enhancing the safety climate through effective near-miss management might reduce workers' intentions to leave the company.

Inadequate response to near-miss reports from workers could elevate turnover intentions among workers. However, to the best of our knowledge, no studies have examined the relationship between companies' responses to near-miss reports and workers' turnover intentions. This study aimed to examine this relationship.

## 2 Materials and methods

### 2.1 Study design and participants

This cross-sectional study was conducted using data from the baseline survey in the Work, Well-being and Safety for Occupational Health Practice and Management Study (W2S-Ohpm Study). This questionnaire-based survey was delivered by an Internet survey company in March 2022. Details of the study protocol have been reported elsewhere [20]. Briefly, target participants were workers in Japan aged ≥20 years. Sampling was conducted to match the proportional representation of gender, age, and regional composition to that of working population of Japan. A total of 29,997 people met the survey criteria. After excluding 2,304 participants who provided invalid responses, 27,693 individuals remained in the baseline.

From the participants, we selected the subjects as follows to fit the aim of this study. We considered full-time, part-time, temporary, and contract employees for employment status, excluding 4,915 participants who were self-employed, family employees, executives, doing a side job at home, and the others. We also excluded 1,516 participants who worked in companies with less than five employees. In addition, we excluded 14,842 participants with no near-miss experiences in the past year and 1,349 who did not report such experiences to their companies. A final total of 5,071 participants were included in the analysis.

This study was approved by the Ethics Committee of the University of Occupational and Environmental Health, Japan (Approval numbers: R3-076). Informed consent was obtained from all participants using an online form provided by an Internet survey company.

### 2.2 Assessment of companies' responses to near-miss reports

To assess companies' responses to near-miss reports, we asked, “Did the company (your supervisor) respond to your near-miss report by investigating the cause or taking measures to prevent the accident?” Respondents selected one of three options: “The company adequately responded through cause investigation and equipment enhancement,” “The company inadequately responded,” or “The company did not respond at all.” We classified these into adequate response group, inadequate response group, and no response group, respectively.

### 2.3 Assessment of turnover intentions

To assess turnover intentions of participants, we asked, “How often have you seriously considered leaving your current job?” They selected one of six options: “never,” “almost never,” “sometimes,” “somewhat often,” “quite often,” and “very often.” We classified those who answered “never,” “almost never,” and “sometimes” as not having turnover intentions, whereas those who answered “somewhat often,” “quite often,” and “very often” as having turnover intentions [21].

### 2.4 Covariates

Covariates comprised sex, age, education, marital status, annual household income, employment status, and company size (the number of employees). We selected these covariates based on previous studies using turnover intentions as an outcome [22,23]. Age was expressed as a continuous variable. Education was classified into three categories: junior high or high school, vocational school or college, and university or graduate school. Marital status was classified into three categories: married, never married, and divorced or widowed. We categorized annual household income into six groups: <4.00 million, 4.00–5.99 million, 6.00–7.99 million, 8.00–9.99 million, 10.00–11.99 million, and ≥12.00 million Japanese yen. Employment status was categorized into four categories: full-time employee, part-time employee, temporary employee, and contract employee. Company size was classified into five categories: 5–9, 10–49, 50–99, 100–999, and ≥1,000 employees.

### 2.5 Statistical analysis

We examined the relationship between companies' responses to near-miss reports and turnover intentions using multilevel logistic regression analyses nested for industries. We classified industries into 20 categories according to Japan's standard industrial classification: agriculture and forestry; fisheries; mining and quarrying of stone; construction; manufacturing; electricity, gas, heat supply, and water; information and communications; transport and postal services; wholesale and retail trade; finance and insurance; real estate and goods, retail and leasing; scientific research, professional and technical services; accommodations, eating and drinking services; living-related and personal services and amusement services; education, and learning support; medical, healthcare and welfare; compound services; services, N.E.C. a; government, except elsewhere classified; industries unable to be classified.

The odds ratios (ORs) and corresponding 95% confidence intervals (CIs) were calculated for a crude model and a multivariable adjusted model. The multivariable model was adjusted for age, sex, education, marital status, annual household income, employment status, and company size.

## 3 Results

The study included 5,071 participants. Table 1 presents the characteristics of participants according to companies' responses to near-miss reports. Of the total 5,071 participants, 3,058 (60.3%) were in the adequate response group, 1,484 (29.3%) were in the inadequate response group, and 529 (10.4%) were in the no response group. The number of women in this study was 2,114 (41.7%), and the mean age of the participants was 41.2 years. As for employment status, the number of full-time employees was 3,861 (76.1%), part-time employees was 810 (16.0%), temporary employees was 110 (21.7%), and contract employees was 290 (57.2%). In terms of company size, the largest group was 100–999 employees for 1,614 (31.8%), followed by 10–49 for 1,577 (31.1%), and ≥1,000 for 763 (15.0%).

**Table 1:** Basic characteristics of the subjects

	Adequate response∗	Inadequate response†	No response‡
No. of participants	3,058	1,484	529
Age (y), mean (SD)	40.9 (12.8)	41.6 (12.3)	41.7 (12.3)
Sex, women	1,340 (43.8%)	596 (40.2%)	178 (33.6%)
Education			
Junior high or high school	728 (23.8%)	330 (22.2%)	162 (30.6%)
Vocational school or college	795 (26.0%)	342 (23.0%)	123 (23.3%)
University or graduate school	1,535 (50.2%)	812 (54.7%)	244 (46.1%)
Marital status			
Married	1,602 (52.4%)	774 (52.2%)	255 (48.2%)
Never married	1,079 (35.3%)	501 (33.8%)	193 (36.5%)
Divorced or widowed	377 (12.3%)	209 (14.1%)	81 (15.3%)
Annual household income (million Japanese Yen)			
<3.99	719 (23.5%)	333 (22.4%)	152 (28.7%)
4.00–5.99	837 (27.4%)	416 (28.0%)	158 (29.9%)
6.00–7.99	652 (21.3%)	351 (23.7%)	91 (17.2%)
8.00–9.99	438 (14.3%)	185 (12.5%)	75 (14.2%)
10.00-11.99	181 (5.9%)	94 (6.3%)	24 (4.5%)
>12.00	231 (7.6%)	105 (7.1%)	29 (5.5%)
Employment status			
Full-time employee	2,329 (76.2%)	1,127 (75.9%)	405 (76.6%)
Part-time employee	500 (16.4%)	224 (15.1%)	86 (16.3%)
Temporary employee	68 (2.2%)	34 (2.3%)	8 (1.5%)
Contract employee	161 (5.3%)	99 (6.7%)	30 (5.7%)
Industries			
Agriculture and forestry	15 (0.5%)	9 (0.6%)	5 (0.9%)
Fisheries	3 (0.1%)	2 (0.1%)	0 (0.0%)
Mining and quarrying of stone	2 (0.1%)	5 (0.3%)	4 (0.8%)
Construction	146 (4.8%)	58 (3.9%)	27 (5.1%)
Manufacturing	519 (17.0%)	270 (18.2%)	109 (20.6%)
Electricity, gas, heat supply, and water	50 (1.6%)	24 (1.6%)	12 (2.3%)
Information and communications	103 (3.4%)	50 (3.4%)	12 (2.3%)
Transport and postal services	178 (5.8%)	91 (6.1%)	37 (7.0%)
Wholesale and retail trade	178 (5.8%)	121 (8.2%)	50 (9.5%)
Finance and insurance	101 (3.3%)	38 (2.6%)	24 (4.5%)
Real estate and goods, retail, and leasing	34 (1.1%)	20 (1.3%)	6 (1.1%)
Scientific research, professional, and technical services	39 (1.3%)	31 (2.1%)	9 (1.7%)
Accommodations, eating, and drinking services	85 (2.8%)	48 (3.2%)	14 (2.6%)
Living-related and personal services and amusement services	48 (1.6%)	22 (1.5%)	13 (2.5%)
Education and learning support	180 (5.9%)	89 (6.0%)	14 (2.6%)
Medical, health care, and welfare	911 (29.8%)	369 (24.9%)	90 (17.0%)
Compound services	23 (0.8%)	12 (0.8%)	6 (1.1%)
Services	208 (6.8%)	122 (8.2%)	62 (11.7%)
Government	191 (6.2%)	72 (4.9%)	22 (4.2%)
Industries unable to be classified	44 (1.4%)	31 (2.1%)	13 (2.5%)
Company size (employees)			
5–9	273 (8.9%)	107 (7.2%)	50 (9.5%)
10–49	923 (30.2%)	468 (31.5%)	186 (35.2%)
50–99	389 (12.7%)	221 (14.9%)	77 (14.6%)
100–999	996 (32.6%)	470 (31.7%)	148 (28.0%)
>1000	477 (15.6%)	218 (14.7%)	68 (12.9%)

SD, standard deviation.

∗ Workers who answered that the company adequately responded through cause investigation and equipment enhancement to their near-miss report.

† Workers who answered that the company inadequately responded to their near-miss report.

‡ Workers who answered that the company did not respond at all to their near-miss report.

Table 2 presents the relationship between companies' responses to near-miss reports and turnover intentions of workers. In crude model, compared with adequate response group, the ORs of turnover intentions were significantly higher in inadequate response group and no response group, at 1.74 (95% CI: 1.51–2.00) and 2.58 (95% CI: 2.12–3.14), respectively. In multivariable adjusted model, compared with adequate response group, the ORs of turnover intentions were significantly higher of inadequate response group and no response group at 1.80 (95% CI: 1.56–2.08) and 2.63 (95% CI: 2.15–3.22), respectively.

**Table 2:** Relationship between companies' responses to near-miss reports and turnover intentions of workers

Group	Crude model				Multivariable adjusted model∗			
	OR	95% CI		*p* value	OR	95% CI		*p* value
Adequate response†	1.00	—	—	—	1.00	—	—	—
Inadequate response‡	1.74	1.51	2.00	<0.001	1.80	1.56	2.07	<0.001
No response§	2.58	2.12	3.14	<0.001	2.63	2.15	3.22	<0.001

Multilevel logistic regression analyses were performed nested for industries.

CI, confidential interval; OR, odds ratio.

∗ Multivariable model adjusted for sex, age, education, marital status, annual household income, employment status, and company size.

† Workers who answered that the company adequately responded through cause investigation and equipment enhancement to their near-miss report.

‡ Workers who answered that the company inadequately responded to their near-miss report.

§ Workers who answered that the company did not respond at all to their near-miss report.

## 4 Discussion

This study aimed to examine the relationship between companies' responses to near-miss reports and turnover intentions of workers. Our results showed that one-third of workers perceived that their companies did not respond to their near-miss reports adequately. Workers who answered their companies did not respond to their near-miss reports were more likely to leave their jobs than those who answered that their companies responded adequately.

In the Basic Survey on Industrial Safety and Health in 2015 in Japan, approximately 25% of workers answered that the company's responses to their near-miss reports were inadequate, and we believe this is an issue that needs to be improved [24]. In our study, the percentages of respondents who answered “the company inadequately responded” and “the company did not respond at all” were 29.3% and 10.4%, respectively, indicating a higher percentage of unsatisfied responses compared to the former public survey. Comparisons are difficult due to the different target populations and conditions of the surveys. The public survey targeted about 18,000 permanent and temporary workers employed at about 14,000 companies randomly selected from companies employing 10 or more permanent workers. Both the public survey and this study confirmed that many workers were not satisfied with companies' responses to their near-miss reports.

Our results suggest that even if companies collect near-miss reports, workers' intentions to leave their companies may increase when companies do not take appropriate action to near-miss reports. There are two possible factors for this mechanism. First, low safety climate might cause higher turnover intentions. Near-miss management can improve safety climate, but inadequate near-miss management, such as not responding to near-miss reports, may decrease safety climate. Workers with lower safety climate perceptions can be unsatisfied with their jobs and less engaged at work and more likely to leave the organizations [15]. Second, if companies do not investigate the causes or improve equipment in response to near-miss reports, the risk of workplace accidents will remain high. In such companies, workers will feel more insecure about working there, which might lead to an increase in their intentions to leave their companies.

Employers need to understand companies' inadequate safety activities can lead to turnover of employees. It is necessary for employers to review companies' near-miss management and establish a system to ensure that workers report near-misses and companies can take appropriate action to the reports. It is also important that workers can recognize their companies have adequately responded to their near-miss reports. If companies respond without workers' involvement, workers may feel that their companies did not respond. Previous studies have noted that it is important for workers to be involved in not only identifying near-miss cases but also analyzing them for increasing worker safety awareness [[25], [26], [27]]. In other words, it is important for companies to analyze the causes and consider improvement measures with workers.

Several limitations in this study should be noted. First, because this is a cross-sectional study, it is difficult to identify causal relationships. In the future, a longitudinal study such as a cohort survey should be conducted. Second, sampling bias exists because we conducted an Internet survey, and workers with access to the Internet were targeted. However, the sampling was designed to be equal to the proportions of gender, age, and region of residence of the worker population in Japan, which is a standard population in Japan [20]. Third, recall bias is possible because we conducted the self-administered questionnaire and asked questions that reflect the past year. As a result, participants' answers may not always be accurate in terms of their near-miss experiences, reports, and company responses. However, experiences such as near-misses are likely to be memorable, so the effect of recall bias is considered to be small. Fourth, because of the self-administered questionnaire, self-report bias is taken into account for interpretation of the result. Fifth, companies' responses to near-miss reports are not measured by a quantitative indicator, so different workers may have different perceptions of the company's responses to near-miss reports. In the future, studies with a quantitative indicator should be conducted. Finally, there is a possibility that the adjustment for confounding factors was insufficient. We determined the covariates based on previous studies regarding turnover intentions, but no other study has examined the relationship between companies' responses to near-miss reports and turnover intentions directly, so there may be unknown confounding factors.

## 5 Conclusion

Our results suggested an association between companies' responses to the near-miss reports and turnover intention of workers. Adequate companies' responses to near-miss reports may improve safety climate in companies and reduce workers' intention to leave their companies. Therefore, it is important not only to collect near-misses but also to analyze them with workers and take measures.

## Author contributions

T.N., K.O., and K.M. conceived the study idea; T.N., K.O., N.A., M.N., and K.M. collected the data; A.Y. designed the analysis, analyzed the data, and wrote the draft of the manuscript. All authors have advised on the data interpretation and have reviewed, edited, and approved the final manuscript.

## Ethical approval

The study was approved by the Ethics Committee of the University of Occupational and Environmental Health, Japan (no. R3-076). Only those who gave their consent completed the questionnaire.

## Informed consent

Informed consent was obtained via the survey form on the website.

## Data availability statement

The data that support the findings of this study are available on request from the corresponding author. The data are not publicly available owing to privacy or ethical restrictions.

## Conflicts of interest

The authors declare no conflicts of interest associated with this manuscript. T.N. reports a research grant from TIS Inc. and personal fees from BackTech Inc., EWEL Inc., and Sompo Health Support Inc., outside the submitted work. K.M. reports research grants from DAIDO LIFE INSURANCE COMPANY, Komatsu Ltd., and HASEKO Corporation, scholarship grants from AORC, BackTech Inc., DAIDO LIFE INSURANCE COMPANY, EWEL Inc., iSEQ Inc., JMA Research Institute Inc., MEDIVA Inc., SMS Co., Ltd., Sompo Health Support Inc., and T-PEC COPRORATION, and personal fees from BackTech Inc. and Sompo Health Support Inc., outside the submitted work.
